# Immediate Tendon Transfer for Functional Reconstruction in Upper Limb Sarcoma Surgery: An Observational Study

**DOI:** 10.31729/jnma.9102

**Published:** 2025-07-01

**Authors:** Basanta Maharjan, Janith Singh, Dipendra Maharjan, Binayak Dhungel, Rishi Ram Poudel, Krishna Jung Sah, Prashanna Dip Karki

**Affiliations:** 1 Department of Orthopedics, KIST Medical College and Teaching Hospital & Nepal Cancer Hospital and Research Centre, Imadol, Lalitpur, Nepal; 2 Department of Orthopedics, National Trauma Center, Mahaboudha, Kathmandu Nepal; 3 Department of Orthopedics, Nepal Army Institute of Health Sciences, Sanobharyang, Kathmandu, Nepal; 4 Ortho-Onco Unit, Bhaktapur Cancer Hospital, Dudhpati, Bhaktapur, Nepal; 5 Department of Orthopedics, Patan Academy of Health Sciences, Lagankhel, Lalitpur, Nepal; 6 Department of Orthopedics, Provincial Hospital Malangawa, Malangawa, Sarlahi, Nepal; 7 Department of Orthopedics, B&B Hospital, Gwarko, Lalitpur, Nepal.

**Keywords:** *sarcoma*, *treatment outcome*, *tendon transfer*, *upper extremity*

## Abstract

**Introduction:**

Upper limb sarcoma surgeries often require sacrificing major nerves to achieve oncologic control, leading to significant functional loss. In low-resource settings where microsurgical reconstruction is limited, immediate tendon transfer offers a practical solution for restoring function. This study evaluates functional outcomes following immediate tendon transfer in patients undergoing nerve-sacrificing sarcoma excision.

**Methods:**

This multicentric observational study was conducted at four tertiary centers in Nepal: KIST Medical College and Teaching Hospital, National Trauma Centre, Bhaktapur Cancer Hospital and Nepal Cancer Hospital and Research Centre. The patients who underwent upper limb sarcoma surgery with immediate tendon transfer between January 2021 and May 2024 were included in the study. Data on demographics, tumor type, nerve involvement, surgical procedures, complications, and functional outcomes (Quick DASH, MSTS, and MRC grading) were collected and analysed using SPSS version 20.

**Results:**

This study included 14 patients, with a mean age of 34.07±17.05 years. There were 9 (64.28%) male, and dominant limb involvement was recorded in 10 (71.42%) patients. Bone sarcomas were present in 3 (21.42%) patients and soft tissue sarcomas in 11 (78.57%) patients. The radial nerve was sacrificed in 8 (57.14%) patients. The mean QuickDASH score decreased from 85.17 at 6 weeks to 11.95 at 12 months. The Musculoskeletal Tumor Society score increased from 8.57 to 25.71, and Medical Research Council grading was >4 in all patients at 12 months.

**Conclusions:**

Immediate tendon transfer is an effective reconstructive strategy to restore function after nerve-sacrificing upper limb sarcoma surgery.

## INTRODUCTION

Upper limb sarcomas present a complex surgical challenge for musculoskeletal onco-surgeons and reconstructive microsurgeons, requiring meticulous planning to achieve a balance between oncological control and preservation of limb function.^1^ The anatomical intricacies of the upper limb, with its dense neurovascular bundles and essential motor units, often necessitate the sacrifice of critical nerves during wide resection of sarcomas to ensure negative margins and reduce the risk of local recurrence and metastases.^2^

With improved diagnostic capacity and earlier detection of sarcomas, limb salvage surgery has become increasingly common.^3^ However, reconstructive options following nerve sacrifice remain limited. ITT offers a pragmatic and costeffective alternative that allows for timely functional restoration without dependence on prolonged nerve regeneration processes.^4,5^ It also avoids the complexity, technical demands, and resource intensity associated with microsurgical nerve reconstruction, making it highly suitable for broader implementation in resource-constrained settings like Nepal.

## METHODS

This was a multicentric, cross-sectional observational study conducted at four major tertiary care centers in Nepal: KIST Medical College and Teaching Hospital, National Trauma Centre, Bhaktapur Cancer Hospital and Nepal Cancer Hospital and Research Centre. The study was conducted following ethical approval from the Nepal Health Research Council (Reference number: 1181), ensuring compliance with national ethical guidelines for research.

The study population included patients diagnosed with histologically confirmed primary or recurrent upper limb sarcomas who underwent wide resection requiring the sacrifice of at least one major nerve specifically radial, posterior interosseous, median, or ulnar nerve, followed by immediate tendon transfer based functional reconstruction in the same operative setting. The recruitment period spanned from January 2021 to May 2024. A census sampling technique was employed to include all eligible cases operated during this time frame. Only those patients who had no pre-existing neurological deficit in the affected upper limb, unrelated to the tumor pathology, and had completed at least 12 months of postoperative follow-up were considered for final analysis. Patients with incomplete documentation, insufficient follow-up, or prior trauma-related nerve injuries were excluded from the study.

Data were extracted from institutional surgical logs, inpatient records, operative notes, and outpatient follow-up charts. Data collection was carried out between January 2025 and April 2025 by the primary investigator in collaboration with designated co-investigators at each participating center. Postoperative follow-up assessments at 6 weeks, 3 months, 6 months, and 12 months were retrieved from clinical documentation and physiotherapy records. These assessments included standardized outcome instruments: the Quick Disabilities of the Arm, Shoulder, and Hand (Quick DASH) questionnaire, the Musculoskeletal Tumor Society (MSTS) scoring system, and Medical Research Council (MRC) muscle strength grading, recorded either by the attending surgeons or physiotherapists. Only patients with complete follow-up data for a minimum of 12 months were included to ensure data reliability and consistency in outcome evaluation.

Surgical procedures were performed by a dedicated team of musculoskeletal oncology known as "TeamSarc Nepal," specialized in limb salvage and reconstruction. The choice of tendon transfer was individualized, based on the tumor location, the sacrificed nerve, availability of donor tendons, and the patient's functional demands. The procedures were performed using standardized techniques, which included:

- Radial nerve palsy: Pronator teres (PT) to Extensor carpi radialis brevis (ECRB) for wrist extension; Flexor carpi radialis (FCR) or Flexor digitorum superficialis (FDS) to Extensor digitorum communis (EDC) for finger extension; Palmaris longus (PL) to Extensor pollicis longus (EPL) for thumb extension.^6^ Posterior interosseous nerve (PIN) palsy: FCR or FDS to EDC; PL to EPL.^7^
- Median nerve palsy: Extensor indicis propius (EIP) or Abductor digiti minimi (ADM) opponensplasty to restore thumb opposition.^8^
- Ulnar nerve palsy: Flexor digitorum profundus (FDP) tenodesis to correct claw deformity; EIP to Adductor pollicis; Extensor pollicis brevis (EPB) to first dorsal interosseous; Lasso procedure for claw correction.^9^

All tendon coaptations were performed using the Pulvertaft weave technique, which is known for ensuring a secure and durable tendon-to-tendon junction and has been shown to withstand early mobilization protocols.^10^ This technique is especially beneficial in oncology patients who may require adjuvant radiotherapy, which can otherwise compromise soft tissue healing and tendon integration. Postoperative care involved wound management and 4 weeks of immobilization in a protective splint or slab, followed by physiotherapy that included progressive strengthening exercises and functional reintegration. Functional outcomes were assessed using standardized tools:

- Quick DASH questionnaire: An 11-item questionnaire that evaluates physical function and symptoms of the upper limb. Patients rate difficulty and severity on a scale from 1 to 5. Scores are scaled from 0 (no disability) to 100 (most severe disability).^11^
- MSTS score: Assesses postoperative limb function across six domains: pain, function, emotional acceptance, hand positioning, dexterity, and lifting ability. Each category is scored from 0 to 5, with a maximum total of 30 points; higher scores indicate better function.^12^
- MRC muscle strength grading: Measures muscle strength on a scale of 0 to 5, where 0 indicates no contraction and 5 indicates normal strength. It is commonly used to assess the functional power of transferred or reinnervated muscles.^13^

Data were analyzed using IBM SPSS Statistics for Windows, version 20 (IBM Corp, N.Y., USA). Continuous variables, such as age and functional scores (Quick DASH and MSTS), were expressed as mean ± standard deviation (SD). Variables like gender, limb dominance, tumor type, nerve sacrificed, and muscle strength (as measured by MRC grading) were reported as categorical data, and descriptive statistics were used to analyze their distribution.

Additionally, we performed a descriptive comparison between two commonly used tendon transfers for radial nerve palsy—FCR to EDC and FDS to EDC focusing on postoperative recovery patterns, range of motion, grip strength, and early complications.

## RESULTS

A total of 14 patients met the inclusion criteria and were enrolled in this study. The mean age of the cohort was 34.07±17.05 years. There were 9 (64.28%) male and 5 (35.71%) female. The dominant upper limb was involved in 10 (71.42%) cases. Regarding tumor characteristics, 9 (64.28%) tumors were localized to the arm, and 5 (35.71%) involved the forearm. Bone tumors were observed in 3 (21.42%) cases, and soft tissue sarcomas in 11 (78.57%) cases. None of the patients had distant metastases at the time of initial diagnosis. A total of 9 (64.28%) patients were referred with recurrent tumors following previous unplanned or incomplete excisions, also known as whoops procedures (Table 1).

**Table 1:** Tumor characteritistics of upper limb sarcoma patient undergoing immediate tendon transfer for functional reconstruction (n=14).

Tumor Location	n(%)
Arm	9(64.28)
Forearm	5(35.71)
Tumor types	
Bone tumors	3(21.42)
Soft tissue tumors	11(78.5)
Distant Metastasis at Presentation	0
Recurrent Tumors (Referred After Whoop's Procedure)	9(64.28)

**Table 2:** Histology of upper limbs sarcoma patient undergoing immediate tendon transfer for functional reconstruction (n=14).

Tumor Type	n(%)
Synovial Sarcoma	4(28.57)
Epitheloid Sarcoma	2(14.28)
Osteosarcoma	2(14.28)
Ewing's Sarcoma	1(7.14)
Myxofibrosarcoma	1(7.14)
MPNST	1(7.14)
ERMS	1(7.14)
Clear Cell Sarcoma	1(7.14)
Leiomyosarcoma	1(7.14)

MPNST= Malignant Peripheral Nerve Sheath Tumor; ERMS= Embryonal Rhabdomyosarcoma

**Table 3:** Nerve sacrifice in upper limb sarcoma patient undergoing immediate tendon transfer for functional reconstruction (n=14).

Nerve sacrificed	n(%)
Radial nerve	8(57.14)
PIN	3(21.42)
Median nerve	2(14.28)
Ulnar nerve	1(7.14)
PIN= Posterior Interosseous Nerve	

The histopathological distribution included synovial sarcoma in 4(28.57%) cases, epitheloid sarcoma in 2(14.28%) cases, and osteosarcoma in 2(14.28%) cases. Other tumor types included Ewing's sarcoma, myxofibrosarcoma, malignant peripheral nerve sheath tumor (MPNST), embryonal rhabdomyosarcoma, clear cell sarcoma, and leiomyosarcoma, each reported in 1 case (7.14%) (Table 2).

Nerve sacrifice was necessary as follows: radial nerve in 8 (57.14%) patients, posterior interosseous nerve (PIN) in 3 (21.42%), median nerve in 2 (14.28%), and ulnar nerve in 1 (7.14%) patient (Table 3). All tendon transfers were performed immediately following nerve sacrifice within the same operative session, with no intraoperative complications reported related to the tendon transfer procedure itself.

The mean QuickDASH score at 6 weeks was 85.17±5.07 and decreased to 11.95±6.20 at 12 months. The mean MSTS score increased from 8.57±2.27 at 6 weeks to 25.71±2.33 at 12 months. Muscle strength graded by MRC was less than grade 3 in all patients at 6 weeks and improved to grade 4 or higher by 12 months (Table 4).

One (7.14%) patient developed superficial wound dehiscence, which resolved with local wound care. Finger and wrist stiffness were observed in 3 (21.42%) patients during early follow-up and improved with physiotherapy.

**Table 4:** Functional outcome measures of upper limbs sarcoma patient undergoing immediate tendon transfer for functional reconstruction (n=14).

Outcome Measures	6 weeks	12 months
QuickDASH	85.17 ±5.07	11.95 ±6.20
MSTS Score	8.57 ±2.27	25.71 ±2.33
MRC Grading	<3 in all	>4 in all

MSTS= Musculoskeletal Tumor Society score; MRC= Medical Research Council grading

One (7.14%) patients with ulnar nerve transfer had residual claw deformity, which was managed with dynamic splinting and hand therapy. There were no reported cases of tendon rupture, deep infection, or reoperation.

## DISCUSSION

This multicentric retrospective study highlights the viability and functional efficacy of ITT for restoring upper limb function following nerve-sacrificing upper limb sarcoma resections. Functional impairment due to nerve sacrifice remains a major challenge in limb salvage surgery, particularly in resource-constrained settings like Nepal, where access to advanced microsurgical nerve repair or transfer techniques is limited.

The ITT offers an immediate mechanical solution to restore key motor functions, circumventing the long and often unpredictable course of nerve regeneration and reinnervation. This approach provides patients with earlier functional recovery, reduced disability, and improved quality of life. The substantial improvement in Quick DASH and MSTS scores observed in our cohort supports the clinical effectiveness of ITT in this population. Additionally, the improved MRC muscle strength grades demonstrate successful functional reanimation of motor units, correlating well with patient-reported outcomes. These findings are consistent with prior literature from trauma and polio populations where tendon transfers have reliably restored function and satisfaction.^5,6^

The use of the Pulvertaft weave technique for tendon coaptation likely contributed to the robust repair strength and allowed early mobilization protocols, thereby minimizing risks of tendon rupture or elongation.^10^ This technique is particularly advantageous in oncology patients who often require adjuvant radiotherapy, which can negatively impact soft tissue healing and tendon integrity.

Among radial nerve palsy patients, the FCR to EDC transfer was the preferred technique in 9 (64.28%) patients, associated with faster recovery, improved tendon excursion, and less postoperative stiffness. The FDS to EDC transfer, performed in 2 (14.28%) patients, was more technically challenging and associated with donor finger stiffness and reduced grip strength. These observations are aligned with the findings of Dawood et al., who demonstrated superior functional outcomes and ease of rehabilitation with FCR transfer.^14^

The prevalence of patients undergoing prior unplanned excisions (whoops procedures) in our study (64.28%) reflects a critical issue in sarcoma management. Unplanned excisions without appropriate oncologic margins increase the risk of local recurrence and necessitate more extensive subsequent resections, often involving nerve sacrifice and complex reconstructive procedures.^15^ This highlights the pressing need for early diagnosis and referral to specialized sarcoma centers to avoid such complications and improve functional outcomes.^16^

Limitations of this study include its retrospective design, small sample size, and lack of a control group, which limit the generalizability of the results. Functional outcome measures used are partly subjective and may be influenced by patient motivation and rehabilitation access variability. Future prospective studies with larger cohorts, longer follow-up, and comparison with microsurgical reconstruction or delayed tendon transfer are warranted to further validate these findings and assess long-term durability and cost-effectiveness.

## CONCLUSIONS

Immediate tendon transfer is an effective option for functional reconstruction in patients undergoing nerve-sacrificing upper limb sarcoma surgery, particularly in settings that lack microsurgical expertise.
